# Spatial and temporal estimation of air pollutants in New York City: exposure assignment for use in a birth outcomes study

**DOI:** 10.1186/1476-069X-12-51

**Published:** 2013-06-27

**Authors:** Zev Ross, Kazuhiko Ito, Sarah Johnson, Michelle Yee, Grant Pezeshki, Jane E Clougherty, David Savitz, Thomas Matte

**Affiliations:** 1 ZevRoss Spatial Analysis, 120 N. Aurora St Suite 3A, Ithaca, NY, 14850, USA; 2 New York City Department of Health and Mental Hygiene, New York, NY, USA; 3 Graduate School of Public Health, Department of Environmental and Occupational Health, University of Pittsburgh, Pittsburgh, PA, USA; 4 Department of Epidemiology, Brown University, Providence, RI, USA

**Keywords:** Air pollution, Birth outcomes, Particulate matter, Nitrogen dioxide, Land use regression, NYCCAS, Temporal adjustment

## Abstract

**Background:**

Recent epidemiological studies have examined the associations between air pollution and birth outcomes. Regulatory air quality monitors often used in these studies, however, were spatially sparse and unable to capture relevant within-city variation in exposure during pregnancy.

**Methods:**

This study developed two-week average exposure estimates for fine particles (PM_2.5_) and nitrogen dioxide (NO_2_) during pregnancy for 274,996 New York City births in 2008–2010. The two-week average exposures were constructed by first developing land use regression (LUR) models of spatial variation in annual average PM_2.5_ and NO_2_ data from 150 locations in the New York City Community Air Survey and emissions source data near monitors. The annual average concentrations from the spatial models were adjusted to account for city-wide temporal trends using time series derived from regulatory monitors. Models were developed using Year 1 data and validated using Year 2 data. Two-week average exposures were then estimated for three buffers of maternal address and were averaged into the last six weeks, the trimesters, and the entire period of gestation. We characterized temporal variation of exposure estimates, correlation between PM_2.5_ and NO_2_, and correlation of exposures across trimesters.

**Results:**

The LUR models of average annual concentrations explained a substantial amount of the spatial variation (R^2^ = 0.79 for PM_2.5_ and 0.80 for NO_2_). In the validation, predictions of Year 2 two-week average concentrations showed strong agreement with measured concentrations (R^2^ = 0.83 for PM_2.5_ and 0.79 for NO_2_). PM_2.5_ exhibited greater temporal variation than NO_2_. The relative contribution of temporal vs. spatial variation in the estimated exposures varied by time window. The differing seasonal cycle of these pollutants (bi-annual for PM_2.5_ and annual for NO_2_) resulted in different patterns of correlations in the estimated exposures across trimesters. The three levels of spatial buffer did not make a substantive difference in estimated exposures.

**Conclusions:**

The combination of spatially resolved monitoring data, LUR models and temporal adjustment using regulatory monitoring data yielded exposure estimates for PM_2.5_ and NO_2_ that performed well in validation tests. The interaction between seasonality of air pollution and exposure intervals during pregnancy needs to be considered in future studies.

## Background

Large, population-based studies of the relationship between air pollution and adverse birth outcomes such as low birth weight present a challenge from an exposure assessment perspective [1,2]. The simultaneous need to determine exposures in relevant time windows as well as the need to characterize variation in exposure associated with the spatial location of maternal residences requires accurate data on both temporal and spatial patterns in air pollutant levels.

Studies of air pollution and birth outcomes commonly rely on continuous monitoring data available from regulatory or other ambient monitors [1,3]. Exposure assessment, for example, often involves assigning concentrations based on the nearest continuous monitor or based on inverse distance weighting of the regulatory monitoring network [4-8]. Estimates of exposure can easily be derived from these monitors for exposure windows of interest and have the further advantage that the measurements from a regulatory monitoring network tend to be collected using consistent methodology and include extensive quality control. Unfortunately, although existing regulatory networks can provide data with high temporal resolution (e.g., daily and hourly data), these networks were generally designed to capture trends in overall ambient concentrations for the community and therefore have sparse geographic coverage within urban areas, which can lead to significant exposure misclassification in epidemiologic studies. Although there is wide variability by locality and time period, few urban monitoring networks capture pollutant concentrations at more than 20 locations.

In order to improve estimates of the spatial variation in air pollutants, several studies investigating the links between exposure and birth outcomes have used regression or emissions dispersion models that can account for patterns in traffic and land use near maternal residences [9-16]. These models may use data from emissions inventories or raw measured concentrations collected in the course of intensive, short-term sampling campaigns and can provide more highly resolved spatial estimates. Dispersion models can also be used to produce temporally resolved estimates of pollutants but require temporally resolved inputs on meteorology and emissions. While daily and hourly data on meteorology is widely available, corresponding detailed data on emissions does not generally exist and must be estimated adding significant uncertainty to modeled predictions. Commonly used vehicle emissions software, for example, estimates hourly emissions using a combination of estimated vehicle miles traveled and weights that assign emissions based on estimates of the fleet’s vehicle type, vehicle age, hour of the day and speed [17]. The regression models often used to assign air pollution exposure, known as land use regression (LUR) models, are generally constructed to estimate exposure for a single time window (e.g., an annual or seasonal average). A limited number of studies have attempted to construct higher temporal resolution estimates by adjusting LUR spatial estimates to reflect regional or citywide temporal trends in pollutants (for examples see [9,10,15,18,19]).

The focus on constructing temporally and spatially resolved estimates of exposure is critical in studies of birth outcomes. These studies are complicated by confounding associated with the seasonal effects of weather and seasonality in births [20,21] as well as the uncertainty associated with which exposure intervals are of most concern [22,23]. As such, birth outcomes studies can benefit from a characterization of how exposure buffer distance (spatial) and averaging time (temporal) affect: (1) the relative contribution of temporal vs. spatial variation to the overall variation of exposure estimates; (2) the correlation between two important combustion-related pollutants, PM_2.5_ and NO_2_; and (3) the correlations between estimated exposures across trimesters.

In order to develop spatially and temporally resolved estimates of exposure and investigate the effects of varying spatial buffer sizes and temporal windows, this study takes advantage of unique data resources in New York City (NYC) to assign exposures to fine particulate matter with aerodynamic diameter of 2.5 micrometer or less (PM_2.5_) and nitrogen dioxide (NO_2_) in two-week windows to the maternal residences of 274,996 births. Initiated in 2007 as part of New York City’s sustainability plan, PlaNYC [24], the New York City Community Air Survey (NYCCAS) has been collecting a suite of combustion-related pollutants since December 2008. With 150 monitors in a 790 square kilometer area, NYCCAS has the most comprehensive geographic coverage of any urban air monitoring network in the U.S. The high spatial resolution of the NYCCAS pollution measurements, combined with the large population size of the city, provides unique opportunities for epidemiological investigations that require geographically and temporally resolved estimates of air pollution exposure. In this paper, we describe the development of spatially and temporally resolved estimates of PM_2.5_ and NO_2_ based on a combination of data from NYCCAS and the local regulatory network. We present the model details, the results of a validation, and characteristics of estimated exposures.

## Methods

### Overview of approach for exposure estimation

In estimating the exposures of pregnant mothers in NYC to PM_2.5_ and NO_2_, we used two sources of air pollution data – one to generate a spatial surface of exposure through LUR models and one to temporally adjust the spatial estimates to match gestational exposure intervals (e.g., trimesters). The first year of NYCCAS monitoring was used in LUR models along with geographic data on emissions and land use to generate a spatial surface. This spatial surface allowed us to estimate an annual average pollutant value at any location in New York City. Two-week average concentrations that correspond to time windows within gestational periods were then computed by temporally adjusting the annual average (spatial) concentrations using a city-wide time series computed from continuous *regulatory* monitors (for examples of other studies using this approach see [9,10,15]). Combining the two sources of data allowed us to capture both spatial and temporal variation in air pollution. In this approach, the relative differences in pollutant concentrations between different spatial locations remain the same but absolute concentrations at all locations rise and fall together as city-wide pollutant concentrations are proportionally modified by temporal variation in city-wide weather conditions.

### Data

#### New York City community air survey data and land use variables

Details on the monitoring network and data collection are described elsewhere [25]. Briefly, as part of NYCCAS, two-week average concentrations of several pollutants at street-level (10–12 feet off the ground) were collected in each of the four seasons at 150 locations in New York City for the period December, 2008 through December, 2010. Logistical considerations precluded monitoring at all 150 sites at the same time. Instead, each of the four seasons was divided into six two-week periods (“sessions”) and 25 monitoring units, randomly assigned, operated in any given two week period (a total of 24 two-week sessions per year). The period of December 2008-December 2009 is referred to here as “Year 1” and was used to develop the spatial models while December 2009-December 2010 (“Year 2”) was used for validation. Annual averages were computed using the four seasonal two-week averages after accounting for temporal variation using the approach outlined in [18,26] and described in Additional file 1. A wide range of traffic and land use-related variables were derived for 15 levels of circular buffer regions (50-1000 m) around NYCCAS monitoring sites using geographic information systems (GIS). These variables included traffic density, truck traffic volume, emissions of residual oil for building heating, tree/grass coverage and others. The full list of variables included is described elsewhere [25,27].

#### PM_2.5_ and NO_2_ data from regulatory monitors

Raw data from the US Environmental Protection Agency’s (EPA) Air Quality System were retrieved for all hours and all days from January 1, 2007 to March 31, 2011 for the five boroughs of New York City and neighboring counties in both New York and New Jersey. Hourly records of PM_2.5_ and NO_2_ were averaged into daily values. Daily averages with fewer than 18 valid hourly values were excluded. We limited the data to daily and hourly records without laboratory-related qualifiers or local event qualifiers (e.g., “Construction/Demolition”, “Unique Traffic Disruption”). We retained data with qualifiers associated with regional events that would likely affect the entire city (e.g., “Wildfire-U.S.”, “Stratospheric Ozone Intrusion”).

#### New York City birth data

Birth date, gestational age at delivery (used to generate estimated conception date) and the geographic coordinates for maternal residences for all births in New York City 2008–2010 were obtained from the Bureau of Vital Statistics, New York City Department of Health and Mental Hygiene. The database initially included a total of approximately 380,000 births and 160,000 unique residential locations. After restricting the data to births with 22–42 weeks of gestation, truncating the data to only include births with conception dates between July 31st, 2007 through March 12, 2010 (to avoid the fixed-cohort bias [23]), singleton births, non-smoking mothers, plausible birth weights, and the exclusion of births with congenital malformations, the total number of births was 274,996 at 138,680 unique locations. After the fixed cohort bias adjustment, the distribution of gestational age was consistent across the estimated conception months, with 25th percentile, median, and 75th percentile of 38, 39, and 40 weeks, respectively, but it was negatively skewed (i.e., fewer births with short gestation lengths), as expected. The maternal residence represents the residence at the time of the birth. No information on residential relocation, commuting patterns, or time-activity behaviors shaping individual exposures during pregnancy was available. This study was reviewed and approved by the Institutional Review Board of the New York City Department of Health and Mental Hygiene

### Analysis

#### Computation of spatial estimates (based on data from NYCCAS) through land use regression models

The modeling approach for development of the spatial models is described in detail elsewhere [27]. Briefly, the annual averages at the 150 NYCCAS monitoring locations were used as the response in multiple regression models developed to assign exposure to maternal residences. Geographic variables of emissions and land use derived using GIS were treated as candidate predictors and were grouped into emissions-based categories and tested for inclusion in a linear regression model using forward stepwise selection. LUR model building was conducted on a randomly sampled subset of 125 NYCCAS monitoring locations and validation was conducted at the remaining 25 sites. The final regression models were extended to account for residual spatial autocorrelation using kriging with external drift (KED). This approach is analogous to generating predictions using regression, then kriging the residuals and adding the results together except that all modeling stages are conducted simultaneously using generalized least squares to ensure correct estimation of the prediction errors [28]. To generate a continuous surface of exposure for visualization and computation of block-level and neighborhood level exposure, the KED models were applied to a regular 100 × 100 meter lattice of points covering all of NYC. For presentation purposes, the maps of the 100 × 100 meter lattice were smoothed using inverse distance weighting.

The final KED models were used to assign *spatial* estimates of exposure to PM_2.5_ and NO_2_ to the birth cohort. Exposure estimates were computed for three different spatial scales: 1) a single estimate at the maternal residence (i.e., a KED estimate at specific XY coordinates); and two estimates that were designed to capture approximate neighborhood exposures including 2) an average of KED estimates at 100 meter grid cells within 300 meters of the mother’s home address (to represent block-level exposure); and 3) an average of KED estimates at 100 meter grid cells within 800 meters (0.5 miles) of the mother’s home address (to approximate the average “walkable-distance” neighborhood exposure [29]).

#### Computation of city-wide temporal trends (based on data from regulatory monitors)

Based on an initial review of the correlations and seasonal patterns in the data and discussions with New York State Department of Environmental Conservation staff we limited the raw PM_2.5_ data to (A) monitors within the five boroughs of NYC (excluding adjacent counties) and (B) monitors using the Federal Reference Method (FRM parameter code 88101). We limited to FRM monitors because monitors collecting continuous hourly data using the tapered element oscillating microbalance method have a known seasonal bias in the relationship with FRM (with the continuous, hourly monitors underestimating PM_2.5_ during colder times of the year) [30]. In total there were 5 NO_2_ and 13 FRM PM_2.5_ monitors that collected data at some point during our study period. The *monitoring objective* category associated with all of these regulatory monitors was “Population Exposure”, indicating that these monitors were meant to measure urban background concentrations relevant to population exposures (as opposed to the impact of a specific pollution source). The monitoring sites tend to be located on top of buildings (~20-30 meters above ground) and away from major emissions sources. To avoid spatio-temporal confounding associated with different monitors operating in different time windows, only monitors with at least 75% monitoring completeness in *all* quarters in 2007 through the first quarter of 2011 were included to cover the 2-week periods that span the exposure estimates for the first conception and last births. Five PM_2.5_ monitors at four different locations (sites) and two NO_2_ monitors at two different locations met our completeness criteria standards.

A city-wide daily average for both PM_2.5_ and NO_2_ was computed by averaging daily values across sites. PM_2.5_ monitoring sites included in the analysis operated on either an every-day schedule (1 site in Queens) or an every-third-day schedule (3 sites, one each in Manhattan, the Bronx and Queens). Although daily concentrations at the Queens site are strongly correlated with the other sites (r = 0.98) concentrations tended to be slightly lower (intercept = 0.89, slope = 0.98). To account for this small difference we adjusted the daily values where only the Queens data was available using the linear relationship between the average of all four sites and the value at the Queens site. All NO_2_ monitors collected data on an every-day schedule. Similar to PM_2.5_, days with data from a single NO_2_ monitor were adjusted based on the relationship between that monitor to the average of both monitors (additional detail is provided in Additional file 1). For both pollutants, days with no monitoring data were treated as missing. Days with data from two or three monitors were strongly correlated with averages from all monitors (r > 0.98) and were averaged without adjustment.

#### Temporal adjustment of spatial estimates – two-week window exposure assignment

Spatial PM_2.5_ and NO_2_ estimates for each pregnancy location (representing a single annual average) were temporally adjusted to generate a series of contiguous two-week averages spanning the duration of each pregnancy. We chose two-week averages as the building blocks of exposure (A) in order to be consistent with the NYCCAS sampling protocol which collected air quality data in two-week integrated samples and (B) in consideration of the fact that shorter time intervals would not improve the precision of estimated exposure when they are averaged for the entire gestation period, trimesters, and the last 6 weeks of pregnancy. Temporal adjustment was conducted by computing a ratio of the city-wide average during the two-week window of interest to the city-wide annual average for the year that corresponds to the year used in the spatial modeling (December 2008-December 2009). The spatial estimate was then multiplied by this ratio to generate the temporally adjusted spatial value. Given that pregnancy duration is generally not perfectly divisible by two-week (14-day) periods, the final two-week window often extends beyond the actual birth date (and, potentially, into the first quarter of 2011). These two-week average exposures during the gestation period are the building blocks of final exposure estimates for the analysis of air pollution and birth outcomes (e.g., trimester averages).

#### Validation of temporal adjustment approach

As a validation, we predicted the raw two-week concentrations at the 150 NYCCAS locations in Year 2 (a total of four predictions at each of 150 locations – one for each season) using the temporal adjustment approach discussed above. The predictions were compared to measured concentrations using R^2^ and the mean absolute percentage error. The 600 two-week averages from Year 2 (December 2009-December 2010) were not included in the spatial or temporal model building and thus provide a good opportunity for validation. We also computed, for comparison, exposure estimates based on a nearest monitor approach whereby we assigned the two-week average measured at the nearest monitor as an estimate of exposure – these results are included in Additional file 1. Additional file 1 also provides detail on the apportionment of spatial vs. temporal variation in the raw Year 2 concentrations.

#### Characterization of spatial and temporal contributions to exposure estimates for birth outcomes

To assess the impact of differing spatial buffers and temporal windows on exposure we estimated PM_2.5_ and NO_2_ concentrations for each birth for several combinations of spatial scale and time window. In particular, spatial exposure estimates were generated at the maternal address as well as within the 300 m and 800 m (0.5 mile) buffers around the maternal address. For comparability with previous birth outcomes studies, exposure estimates at each of these spatial scales were computed for five distinct time windows of interest – the last six weeks of gestation, each of the trimesters and the entire gestation period for each birth [31-36]. Each of the estimates reflects a different level of spatial or temporal smoothing between the extremes of purely spatial (i.e., the regression model estimate at the maternal address or buffer region with no temporal adjustment) and purely temporal (i.e., estimates based solely on the city-wide time series with no distinction for the location of the maternal residence). To characterize the temporal and spatial contributions to overall variation the estimated exposures were regressed on the citywide average pollution levels. We compared correlation between PM_2.5_ and NO_2_ in each of these combinations of buffer distance and averaging periods and we examined the correlation between the estimated exposures. Because our exposure estimation is based on two-week blocks of data, the trimesters are defined as follows in gestation weeks: 1st trimester – 1 through 12; 2nd trimester – 13 through 26; 3rd trimester – 27+. For those pregnancies that had incomplete (<37 weeks) 2nd and 3rd trimesters (0.3% and 9%, respectively), the trimester averages are the average of available two-week block averages.

## Results

### Spatial estimates

Across all NYCCAS sampling sites in Year 1, annual PM_2.5_ at street level averaged 11.3 μg/m^3^ (standard deviation = 2.1 μg/m^3^). The geographic differences in annual average PM_2.5_ concentrations were most strongly associated with nearby truck traffic and with the density of boilers burning residual heating oil (#4 or #6 grade). The final regression model includes the average density of truck traffic within 1600 meters (1 mile), the number of boilers burning residual oil within 1 kilometer, the area of industrial land use within 500 meters, the land area with vegetative cover within 100 meters (an inverse association; more vegetative cover was associated with less PM_2.5_) and overall traffic weighted road density within 100 meters (Table 1). The final spatial, LUR model (before KED) predicted the 25 validation locations, locations that were not part of the original modeling, to within 6% of true values (R^2^ = 0.85). The validation samples were returned to the pool of modeling samples and the residual spatial autocorrelation was estimated. The model exhibited modest residual spatial autocorrelation. A review of the variogram cloud indicated that three locations had a disproportionate effect on the variogram (these sites had unusually large residuals in comparison to nearby sites) and were excluded from variogram fitting. These sites were only removed to fit the variogram, the final regression model and final KED model are based on all 150 locations. The final empirical variogram was fit with an exponential variogram model with a range of 5.5 kilometers. In order to capture the smooth regional patterns exhibited in the residuals, the variogram was fit without the first variogram bin (i.e., pairs separated by an average of 0.5 miles were excluded from the variogram fitting). The overall variation explained by the KED model with all samples based on the squared correlation between raw and fitted values is 79%.

**Table 1:** Land use regression coefficients from the model using kriging with external drift (KED), including the variogram fit

**Fine particulate matter model variables**	**Beta**	**Std. error**	**t-value**	**p-value**		
(Intercept)	10.03	0.28	35.45	<0.01	**Exponential variogram model**	
Industrial land use within 500 m	5.05	1.67	3.02	<0.01	Range (KM)	5.53
Number of boilers burning residual oil within 1 km	0.01	0.00	7.68	<0.01	Partial Sill	0.36
Average density of truck traffic within 1.6 km	0.16	0.06	2.85	0.01	Nugget	0.52
Estimated overall traffic weighted road density within 100 m	0.01	0.00	6.10	<0.01		
Land area with vegetative cover within 100 m	−57.60	11.43	−5.04	<0.01	**Overall R-sq**	0.79
**Nitrogen dioxide model variables**	**Beta**	**Std. error**	**t-value**	**p-value**		
(Intercept)	21.11	1.25	16.89	<0.01	**Spherical variogram model**	
Interior square footage of buildings within 1km	0.92	0.10	9.61	<0.01	Range (KM)	18.84
Nighttime population within 1 km	0.00	0.00	1.55	0.12	Partial Sill	3.71
Estimated overall traffic weighted road density within 100 m	0.02	0.00	4.26	<0.01	Nugget	8.15
Location on a bus route (Categorical)	4.94	0.69	7.16	<0.01		
Land area with vegetative cover within 100 m	−309.98	47.76	−6.49	<0.01	**Overall R-sq**	0.80

Annual NO_2_ averaged 27.2 ppb in Year 1 (standard deviation = 8.8 ppb) across NYCCAS sites. Differences in NO_2_ between locations were most strongly associated with density of built space within 1 kilometer of the sampling site and the amount of traffic within 100 meters. The final model includes interior square footage of buildings within 1 kilometer, traffic density within 100 meters, vegetative cover within 100 meters (an inverse association), location on a bus route (dichotomous) and nighttime population within 1 kilometer. The final spatial, LUR model (before KED) predicted the 25 validation locations, locations that were not part of the original modeling, to within 13% of true values (R^2^ = 0.74). If one validation site with an NO_2_ value of 59 ppb is removed the R^2^ increases to 0.80. The validation samples were returned to the pool of modeling samples and the residual spatial autocorrelation was estimated. Similar to PM_2.5_ the model exhibited modest residual spatial autocorrelation. Three locations with a disproportionate effect on the variogram were excluded and the final empirical variogram was fit with an exponential variogram model with a range of 11.7 kilometers. Similar to the PM_2.5_ models the three sites were removed only for variogram fitting and the final regression model and KED model use the full 150 sites. The significance of the nighttime population variable in the KED was diminished (p < 0.15) compared with the multiple regression model but was retained due to the strength of the variable in the regression. The overall variation explained by the KED model with all samples is 80%.

**Figure 1:**
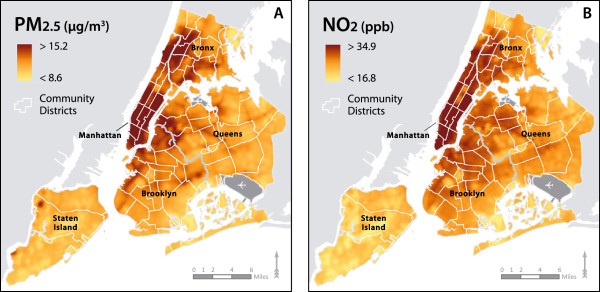
**Map of spatial (KED) estimates for PM**_**2.5 **_**and NO**_**2**_**.**

Based on the spatial surfaces of pollutant concentrations (Figure 1A, B), both PM_2.5_ and NO_2_ exhibit similar geographic patterns with higher concentrations in Manhattan and lower concentrations in Staten Island. Southern areas of the Bronx also exhibit relatively high concentrations for both pollutants while coastal areas have lower concentrations.

The regression-based models were used to generate “spatial” predictions at maternal residences and on a regular 100 m × 100 m lattice from which we derived the 300 m and 800 m buffer average estimates.

### Computation of city-wide temporal trends

**Figure 2:**
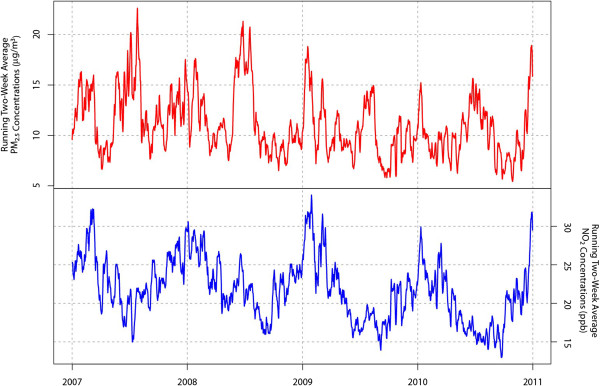
**Time series for PM**_**2.5 **_**and NO**_**2**_**.**

Among the regulatory monitoring data there were five PM_2.5_ FRM monitors at four different NYC locations (a single site can have multiple monitors) that met our completeness criterion for the data analysis period. In total 0.5% of observations were removed due to EPA database qualifiers. The measurements from the two monitors with complete data at the same monitoring site were highly correlated across all days (r = 0.99) and were averaged within the site. Sites with complete data include a site in northern Manhattan, the Bronx, Queens and Staten Island providing good overall spatial coverage (site details can be found in Additional file 1). All four sites are strongly correlated across the entire time period with bivariate correlation based on daily values ranging from 0.85 to 0.95, providing evidence of a consistent citywide temporal trend. Missing data was imputed (details in Additional file 1) and daily values were averaged. PM_2.5_ concentrations tend to be elevated both in summer and winter (Figure 2). The bi-annual cycle of the PM_2.5_ temporal pattern reflects alternate contributions from summer-time chemical constituents (e.g., sulfate) and winter-time chemical constituents (nitrate) to the total PM_2.5_ mass.

There were two monitors with complete NO_2_ data, both collecting data every day – one site in Queens and one in the Bronx. No observations were removed due to EPA database qualifiers. Both sites had complete data for all quarters at the 75% threshold except for a single quarter at the site in the Bronx (at this site, the third quarter of 2007 was complete at 68%). We opted to include this site in the computation of the citywide temporal trends despite the single quarter slightly below 75% completeness. Missing data was imputed (details in Additional file 1) and daily values were averaged. Daily values at the two NO_2_ sites are strongly correlated across the entire time period (r = 0.88). Concentration peaks occur in the winter (and troughs in the summer) for NO_2_ (Figure 2). The winter peaks likely reflect both the lower mixing heights (i.e., less atmospheric mixing and ventilation) and increased emissions from oil burning for heating.

### Temporal adjustment of spatial estimates

The ratios of two-week (14 day) averages to the yearly average used in the NYCCAS Year 1 modeling for citywide concentrations ranged from 0.52 to 2.19 for PM_2.5_ and 0.60 to 1.58 for NO_2_. These ratios were used to adjust the spatial estimates at the maternal residences and generate contiguous two-week averages throughout the gestation period. For validation purposes, the ratios were also applied similarly at NYCCAS monitoring locations to produce two-week predictions corresponding to the two week monitoring periods in Year 2.

### Validation of temporal adjustment approach (application to Year 2 NYCCAS data)

**Figure 3:**
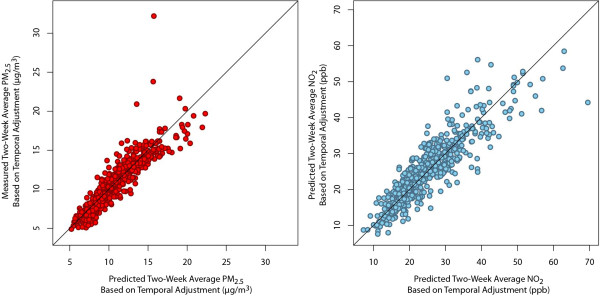
**Comparison of measured PM**_**2.5 **_**and NO**_**2 **_**concentrations vs predictions using the temporal adjustment method.**

In Year 2 measured two-week average PM_2.5_ at NYCCAS sites ranged from 4.9 to 32.2 μg/m^3^ (<1% missing values) and NO_2_ ranged from 7.6 to 58.5 ppb (1% missing values). Approximately 56% of variation in the raw PM_2.5_ concentrations and 18% for NO_2_ is attributable to temporal variation (details in Additional file 1). The temporal adjustment method was used to generate predictions of the 600 two-week concentrations of PM_2.5_ and NO_2_ from Year 2. Predictions for both pollutants were strongly correlated with measured concentrations (Figure 3). For PM_2.5_ the R^2^ for predicted vs. actual concentrations (597 non-missing two-week averages) was 0.83 (0.88 if two high concentrations observations are removed) with a mean absolute percentage error of 8%. With the two high concentration sites removed, the season-specific R^2^ are similar to each other and range between 0.81 and 0.87 (including the two sites decreases the spring R^2^ to 0.73). For NO_2_ the overall R^2^ (594 non-missing two-week averages) was 0.79 with a mean absolute percentage error of 12%. NO_2_ predictions were less precise during the winter (R^2^ = 0.72) than for the other three seasons (R^2^ 0.83-0.88) and this pattern is not attributable to a small number of predictions.

### Characterization of spatial and temporal components of exposure estimates at maternal residences

For each pollutant, the spatial-only (non-temporally adjusted) exposure estimates at the three spatial scales (maternal residences and two buffer levels) were highly correlated (r: 0.95 to 0.99 for PM_2.5_; r: 0.86 to 0.98 for NO_2_). The spatial only correlation between the two pollutants ranged from 0.79 at the maternal address to 0.88 at the 800 m buffer distance.

**Figure 4:**
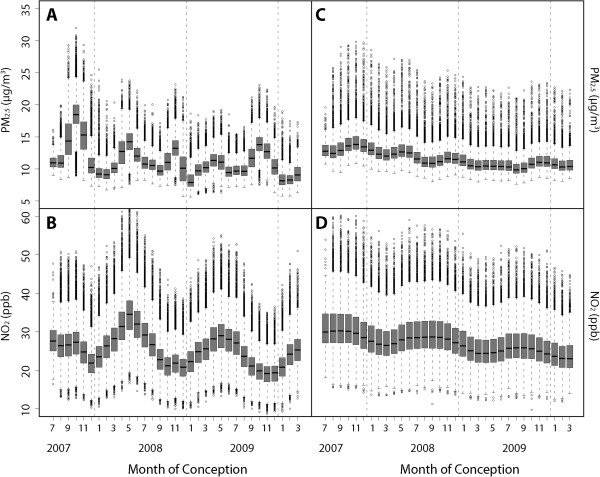
**Box plots describing distribution of: estimated PM**_**2.5 **_**(A) and NO**_**2 **_**(B) exposures over the last 6 weeks of gestational period, averaged over a 0.5-mile buffer distance from maternal address, displayed by conception month; and, estimated PM**_**2.5 **_**(C) and NO**_**2 **_**(D) exposures over the entire gestational period, at maternal address, displayed by birth month.**

The relative contribution of temporal and spatial variation to the estimated exposures varied between the two pollutants, the exposure interval used and, to a lesser extent, the spatial scale (Table 2). As expected, larger buffer sizes (i.e., more averaging of spatial variation) diminished the contribution of spatial variation to overall variation, though the magnitude of its impact was not substantial. Likewise, longer averaging time windows resulted in a smaller contribution from temporal variation. To illustrate the contrasts, Figure 4 shows box plots of the distribution of the estimated exposures in the birth cohort, sorted by the estimated month of conception using two extreme combinations of buffer scale/exposure averaging time windows from Table 2 (note that the distribution in the first box, July 2007, appears narrow because the cut-off for the adjustment for the fixed cohort bias, July 31st 2007, made all the births in this conception month to be on the same day, restricting time-window variations across births). Figure 4A, B show the distribution of estimated exposures by conception month using the largest buffer distance (800 m) and the shortest exposure averaging window (the last 6 weeks of gestation period), a combination that maximizes the temporal variation. Figure 4C, D show the distribution of exposures using the combination of maternal address and the longest exposure averaging window (the entire gestation period) a combination that minimizes the temporal variation. On the whole, Figure 4 and the higher R^2^ values for PM_2.5_ in Table 2 indicate that temporal variation contributes more to overall variation for PM_2.5_ than NO_2_.

**Table 2:** **Amount of the overall variation (R**^**2**^**) explained by temporal patterns using varying buffers and averaging exposure interval**

	**First trimester**	**Second trimester**	**Third trimester**	**Last 6 weeks**	**Entire gestation**
**PM2.5**					
Maternal address	0.60	0.56	0.56	0.73	0.34
300 m	0.65	0.61	0.61	0.77	0.39
800 m	0.67	0.63	0.63	0.78	0.41
**NO2**					
Maternal address	0.32	0.35	0.36	0.42	0.14
300 m	0.34	0.38	0.38	0.44	0.15
800 m	0.35	0.38	0.39	0.45	0.16

The within-pollutant correlations of estimated exposures across trimesters are influenced by the pollutant’s temporal (seasonal) variation. Table 3 shows the correlations of the estimated exposures across trimesters and the entire gestation period for PM_2.5_ and NO_2_ at the three buffer levels. For PM_2.5_ the estimated exposures in adjacent trimesters (the 1st and 2nd; the 2nd and 3rd) are weakly correlated (r = 0.23 to 0.32), but those for the 1st and 3rd trimesters are more strongly correlated (r = 0.73 to 0.76), likely because the 1st and 3rd trimesters fall close to peaks/troughs of the bi-annual cycle of PM_2.5_’s temporal pattern. In contrast to the PM_2.5_ result, for NO_2_, the correlations for the estimated exposures in the adjacent trimesters (r = 0.66 to 0.70) are higher than those between the 1st and 3rd trimesters (r = 0.44 to 0.48), likely because the annual cycle of NO_2_’s temporal pattern make adjacent trimesters’ levels more similar. Averaging pollutant concentrations within 3 different buffers around each residence did not substantively change exposure estimates.

**Table 3:** Within-pollutant correlations (Pearson’s r) between different temporal averaging windows and spatial scales

	**PM**_**2.5**_			**NO**_**2**_		
	**1st trimester**	**2nd trimester**	**3rd trimester**	**1st trimester**	**2nd trimester**	**3rd trimester**
Maternal address						
2nd Trimester	0.32	-	-	0.70	-	-
3rd Trimester	0.76	0.32	-	0.48	0.69	-
Entire gestation	0.85	0.69	0.86	0.83	0.92	0.84
300-meter buffer						
2nd Trimester	0.26	-	-	0.69	-	-
3rd Trimester	0.74	0.26	-	0.45	0.67	-
Entire gestation	0.84	0.66	0.84	0.81	0.92	0.83
800-meter buffer						
2nd Trimester	0.24	-	-	0.68	-	-
3rd Trimester	0.73	0.23	-	0.44	0.66	-
Entire gestation	0.84	0.65	0.84	0.81	0.92	0.83

Table 4 summarizes correlations between the estimated exposures to PM_2.5_ and NO_2_ for all the combinations of the averaging time windows and spatial buffers. The correlations are the largest when the exposures were averaged for the entire pregnancy periods and the smallest for the average of the last 6-weeks of gestation. In other words, the common spatial variation increases the correlation between the two pollutants, and the seasonal variations (bi-annual for PM_2.5_ and annual for NO_2_) reduce the correlation.

**Table 4:** **Correlations (Pearson’s r) between PM**_**2.5 **_**and NO**_**2 **_**for varying buffers and averaging exposure interval**

	**First trimester**	**Second trimester**	**Third trimester**	**Last 6 weeks**	**Entire gestation**
Maternal address	0.60	0.56	0.50	0.45	0.76
300 m	0.63	0.59	0.52	0.46	0.81
800 m	0.62	0.58	0.51	0.45	0.81

## Discussion

This study describes and validates an approach for assigning prenatal exposure estimates to PM_2.5_ and NO_2_ in a birth outcomes study based on temporally adjusting spatial estimates from a land use regression model. Reliance on sparse regulatory monitoring networks has significantly constrained the ability of previous studies of birth outcomes to accurately capture geographic variation in prenatal exposure [5,10]. Several recent studies have been able to take advantage of non-regulatory monitoring networks to vastly expand geographic coverage [9,37], but NYCCAS, with 150 monitors in an area of 790 square kilometers, has significantly higher density than previous birth outcomes studies in major urban areas with a very large number of births available for analysis. This monitor density afforded a unique opportunity to capture geographic variation in PM_2.5_ and NO_2_ in the largest city in the US.

We found that the temporal adjustment approach predicted measured values in the validation well. We further found that the overall variation in PM_2.5_ is more strongly influenced by temporal variation than NO_2_. This likely reflects differences in sources of these pollutants. A significant percentage of PM_2.5_ concentrations originates from non-local sources (e.g., transported sulfate) and blankets the city relatively evenly reducing spatial variation [38]. The larger local contribution to NO_2_ by traffic and oil burning, on the other hand, results in greater overall spatial variation. The extent of the temporal contribution to the overall exposure variation, correlation between the two pollutants and correlations across trimesters varied depending on the averaging time window of exposures. The three spatial buffers made only a small difference in the parameters examined. These results are useful in interpreting results from a health effects analysis and in comparing the results from the study using these estimates to previous research.

Implicit in adopting this temporal adjustment approach is the assumption that relative spatial differences in pollutant levels remain constant across the time windows relevant to birth outcomes studies (e.g., trimesters) [1]. The high site-level correlation between concentrations in different seasons and years of monitoring provides strong evidence for this assumption. For example, the correlation between annual concentrations of PM_2.5_ and NO_2_ at NYCCAS locations in Year 1 compared with concentrations at these same locations in Year 2 is 0.93 (Pearson’s r) and 0.96, respectively. In addition, site-level correlations between the 8 seasonal concentrations average 0.81 for PM_2.5_ and 0.88 for NO_2_ with no season-to-season correlation falling below 0.72. Finally, the strong results from the validation – predicting the 600 two-week averages from Year 2 – also provides evidence for the consistency of the spatial pattern.

For spatial scale, we made an *a priori* decision to consider three levels: maternal residential address, 300 m buffer, and 800 m (0.5 mile) buffer from the maternal address. The effect of these spatial buffers on the exposure estimates was observable but not substantial when compared to the averaging time window. Thus, we expect that the results of health effects analyses would not be especially sensitive to the choice of spatial buffer in assigning exposures among the three levels used in this study.

In our study, the correlation between PM_2.5_ and NO_2_ varied depending on the averaging time and trimester. The highest correlation between the two pollutants occurred when the exposures were averaged over the entire gestation period, which would minimize the temporal correlation and maximize the spatial correlation. In the context of multi-pollutant assessment of the health effects, our results suggest that the health effects analysis will need to consider how the averaging time (or buffer) can alter correlations among pollutants and can influence examination of confounding.

Past birth outcome studies that examined multiple pollutants indicated that the trimester with the strongest association varied across pollutants [32-34,39]. Based on our results, it is conceivable that these differences in the trimester-specific associations across pollutants are due to their difference in seasonal patterns (which can vary from region to region). If the biologically relevant exposure is a longer time period, then spatial variation is the larger part of overall variation; if the biologically relevant exposure is a shorter time period, then the model needs to capture such temporal variation. However, given our result that the relative contributions of spatial and temporal variation to the overall variation of estimated exposures change depending on the averaging time window and buffer size, it is also possible that the relative influence of confounding by spatial factors (e.g., socio-economic status) and temporal factors (e.g., seasonality) can change depending on the buffer size and averaging time of the data analytical design. Thus in future epidemiological studies of birth outcomes the analytical design will need to consider characteristics of potential spatial and temporal confounders and plan sensitivity analyses accordingly to better interpret results.

There are several important limitations to this analysis. First, the requirement that we use two different sources of air monitoring data – one to capture spatial patterns and one for temporal patterns – restricted our capacity to evaluate possible changes in geographic patterns through time. Although the validation described above and the comparison of NYCCAS data through time provide evidence for a consistent spatial pattern, localized variation in weather and changes in land use or traffic patterns could have resulted in some variation in the spatial pattern through time that was not captured in this analysis. Second, the birth data includes no details on residential mobility and time activity patterns. An assumption behind our exposure assignment, therefore, is that the concentrations at and near maternal residential locations were representative of exposures experienced during gestation. Mothers who move or spend significant time away from their residential location may be misclassified and the potential for misclassification associated with mobility will be highest for the first and second trimesters when moves are more likely to occur [40]. These issues need to be considered when interpreting the results. Third, only five ambient continuous NO_2_ monitors operated at any point in the four year window and just two of these collected complete data during the study period. Although these two monitors are separated by 15 km and have different land use and traffic patterns the limited number and geographic coverage provided by the NO_2_ monitors restricts our capacity to assess the consistency of the temporal patterns across the city. A previous study, however, found that the median monitor-to-monitor daily correlation of NO_2_ across 17 NYC metro area monitors was 0.87 [41], suggesting that the limited number of regulatory monitors is not a serious problem for the temporal adjustment method we applied. Finally, unique aspects of this analysis may preclude using the methods in other locations or for other pollutants. The methods, for example, require a geographically dense monitoring network as well as regulatory monitoring network with complete data across the time period of interest. In addition, for pollutants without a consistent city-wide temporal trend (e.g., more localized or sparse sources) the temporal adjustment approach may not be appropriate.

## Conclusions

We assigned exposure estimates for PM_2.5_ and NO_2_ to maternal residences for a birth cohort in New York City. Contiguous two-week average concentrations spanning each pregnancy were computed by temporally adjusting a spatial surface based on monitoring from the New York City Community Air Survey, one of the largest urban air monitoring networks in the country. The methodology yielded good predictions in a validation analysis. The resulting estimated PM_2.5_ exposures for the births generally exhibited stronger temporal variations than for NO_2_. The differing seasonal patterns in these two pollutants result in varying correlations in the estimated trimester exposures. The complexity of the interaction between the seasonality of air pollution and the exposure interval during pregnancy will need to be taken into consideration in the interpretation of the health effects analyses in future studies of birth outcomes.

## Abbreviations

PM2.5: Fine particulate matter; NO2: Nitrogen dioxide; NYCCAS: New York City Community Air Survey; GIS: Geographic information systems; LUR: Land use regression; KED: Kriging with external drift; FRM: Federal reference method (for monitoring PM_2.5_).

## Competing interests

The authors declare that they have no competing interests.

## Authors’ contributions

ZR and KI conducted much of the analysis and wrote the manuscript. SJ, MY and GP contributed data analysis, data preparation and manuscript preparation. JC contributed to the analysis methods and manuscript preparation. DS and TM conceived of and managed the project and helped prepare and critically analyze the manuscript. All authors read and approved the final manuscript.

## Supplementary Material

Additional file 1 Additional detail on monitoring sites and the nearest monitor approach. Click here for file
