# Supplementary material for: Spatial and temporal estimation of air pollutants in New York City: exposure assignment for use in a birth outcomes study
Source: Environ Health. 2013 Jun 27;12:51. doi: 10.1186/1476-069X-12-51 (PMC3704849; doi:10.1186/1476-069X-12-51)
Supplement: Additional file 1 — Additional detail on monitoring sites and the nearest monitor approach. [file 1476-069X-12-51-S1.docx]

**Additional file 1**

**Additional details on the computation of the annual averages at NYCCAS locations**

Details on the monitoring network and data collection are described elsewhere [1]. Briefly, as part of NYCCAS, two-week average concentrations of several pollutants at street-level (10-12 feet off the ground) were collected in each of the four seasons at 150 locations in New York City for the period December, 2008 through December, 2010. The period of December 2008-December 2009 is referred to here as "Year 1" and was used to develop the models while December 2009-December 2010 ("Year 2") was used for validation. Budgetary limitations precluded monitoring at all 150 locations simultaneously for the full time period. As a result, 25 sites selected randomly (stratified by boroughs split into east and west) were monitored in each two-week period ("session"). However, the two-week samples collected at the 150 sites are influenced by weather-driven citywide general pollution levels occurring during the two-week sessions. Therefore, the two-week samples from five continuous reference sites were used to temporally adjust each of the two-week samples from the 150 sites. These five reference sites are centrally located in each of five boroughs and are away from major pollution sources and were not included in the modeling. Thus, in each season, each two-week sample value was multiplied by the ratio of the seasonal mean of the reference sites to the mean of that two-week session across the reference sites. Then, the four temporally-adjusted seasonal two-week averages in Year 1 were averaged to generate annual averages, resulting in the spatial NYCCAS data, which are the dependent variable in a land-use regression.

**Details on which regulatory sites were included in the computation of the city-wide temporal pattern and the imputation of missing data**

Sites with complete data include a site in northern Manhattan (AQS ID 360610079, every-third-day schedule), southern Bronx (AQS ID 360050080, every-third-day schedule), Queens (AQS ID 360810124, every-day schedule), and Staten Island (AQS ID 360850067, every-third-day schedule) providing good overall spatial coverage. There were two monitors with complete NO_2_ data, both collecting data every day – one site in Queens (AQS ID 360810124) and one in the Bronx (AQS ID 360050133).

**Handling Missing Regulatory Air Quality Data**

Some PM_2.5_ regulatory monitors used in this study operate on different monitoring schedules. The one site in Queens operated every day and the other three sites operated every three days. Although the four sites are strongly correlated the values at the Queens site are slightly lower. To account for this difference we adjusted the values on days with data from only the Queens monitor. The adjustment was conducted using the linear relationship between the value at the Queens site and the four-site average for days with data from all four sites (i.e., AvgOfAllMonitors~ValueAtQueensSite, yielding an R^2^ of 0.96, a slope of 0.98 and an intercept of 0.89). Fifty-two days (3%) had no monitoring data and were not imputed. The mean citywide daily PM_2.5_ value in Year 1 was 11.0 µg/m^3^ (1st quartile =6.4, 3rd quartile = 14.1, min=2.5, max=42.2).

For days with missing data from one NO_2_ monitor, the two-monitor average was imputed using the linear relationship between the site with missing data and the two-monitor average for days with data from both monitors. One of two regression formulas would be used depending on which value was missing. Formula 1 would be AvgOfBothMonitors~ValueAtQueensSite (R^2^ of 0.94, intercept=1.47, slope 0.95) and Formula 2 would be AvgOfAllMonitors~ValueAtBronxSite (R^2^ of 0.94, intercept=1.15, slope 0.93). For NO_2_, the overall daily mean was 21.8 ppb (1st quartile =16.0, 3rd quartile = 26.3, min=5.0, max=57.5).

**Details on the breakdown of spatial and temporal variation in the Year 2 measured concentrations**

Similar to the approach outlined in [2] we used a mixed model approach to apportion the spatial and temporal variation in the raw concentrations from Year 2 using the R package lme4 (function lmer). The model setup includes the raw concentrations as the response, no predictor variables and the monitoring sessions as random effects (which differs slightly from [2]). In this setup within session variability would represent a purely spatial signal (i.e., within a single monitoring session there will be no temporal variation between locations) and the residual variation would approximately represent the temporal signal (plus measurement error and any residual spatial variation associated with different monitors operating in different sessions). Based on this approach 56% of overall variation in PM_2.5_ is temporal and 44% is spatial. For NO_2_ 18% of overall variation is temporal and 82% is spatial. Note a nearly analogous approach – regressing the raw concentrations on the city-wide temporal patterns (which does not account for non-independence associated with repeated measurements at the same site) – yields a similar estimate for the temporal contribution (54% for PM_2.5_ and 18% for NO_2_).

The mixed modeling approach is appropriate in the context of the validation of Year 2 concentrations because we have repeated measurements at individual locations. For the predictions at maternal residences, however, we do not have repeated measures. Although there are multiple estimates at a maternal address they are not mutually exclusive (the six week estimate is within the final trimester which is within the full gestation period) and they would be used individually in health models. Therefore, the breakdown of spatial and temporal variation in the maternal residence predictions is done separately for each estimate by regressing the predictions against the city-wide time series' and presented in Table 3.

**Details on a comparison of the temporal adjustment approach against the "nearest monitor" approach**

For comparison with the temporal adjustment approach we computed exposure estimates based on the concentrations at the nearest monitor. The nearest monitor approach is common in air pollution epidemiology and we felt it would provide a useful comparison. For the purposes of the nearest monitor calculations, the monitors were not limited to monitors with complete data across the four years – instead all NO_2_ and PM_2.5_ (FRM) monitors with complete data during a particular two-week session were included. The predictions were compared to measured concentrations using R^2^ and mean absolute percentage error

Predictions of two-week concentrations from the two approaches (temporal adjustment and nearest monitor) at validation locations in Year 2 were strongly correlated for PM_2.5_ (r=0.77 for Year 2). The two approaches were less correlated for NO_2_ (r=0.60).

For both PM_2.5_ and NO_2_ predictions using the temporal adjustment approach outlined in the main paper were significantly better than the nearest neighbor approach (Table 1). Temporal adjustment, on average yielded about half the mean absolute percentage error as the nearest monitor approach and resulted in 2-3 times more predictions within 5% of actual values. The R^2^ value for predictions of PM_2.5_ against actual concentrations using the nearest neighbor was 0.52 compared to 0.83 for proportional adjustment for Year 2 (Figure 3). For NO_2_ the R^2^ value for Year 2 using nearest monitor was 0.23 compared to 0.79 for nearest monitor.

**References**

1. Matte TD, Ross Z, Kheirbek I, Eisl H, Johnson S, Gorczynski JE, Kass D, Markowitz S, Pezeshki G, Clougherty JE: **Monitoring intra-urban spatial patterns of multiple combustion air pollutants in New York City: Design and implementation**. *Journal of Exposure Science and Environmental Epidemiology* 2013, **23**:223–231.

2. Johnson M, Macneill M, Grgicak-Mannion A, Nethery E, Xu X, Dales R, Rasmussen P, Wheeler A: **Development of temporally refined land-use regression models predicting daily household-level air pollution in a panel study of lung function among asthmatic children.** *Journal of Exposure Science & Environmental Epidemiology* 2013, **23**:259–267.

**Additional File: Table 1 – comparison of validation predictions using the proportional and nearest monitor approach.**

| **Pollutant** | **Method** | **n** | **MAPE** | **Intercept** | **Slope** | **R-squared** | **Within 5%** | **Within 10%** | **Within 20%** |
| --- | --- | --- | --- | --- | --- | --- | --- | --- | --- |
| PM2.5 | Proportional Adjustment | 597 | 0.08 | 0.45 | 0.95 | 0.83 | 0.42 | 0.70 | 0.93 |
| PM2.5 | Nearest FRM Monitor | 597 | 0.15 | 1.44 | 0.94 | 0.52 | 0.22 | 0.41 | 0.69 |
|  |  |  |  |  |  |  |  |  |  |
|  |  |  |  |  |  |  |  |  |  |
|  |  |  |  |  |  |  |  |  |  |
| **Pollutant** | **Method** | **n** | **MAPE** | **Intercept** | **Slope** | **R-squared** | **Within 5%** | **Within 10%** | **Within 20%** |
| NO2 | Proportional Adjustment | 594 | 0.12 | 3.38 | 0.87 | 0.79 | 0.30 | 0.53 | 0.83 |
| NO2 | Nearest Monitor | 594 | 0.25 | 6.07 | 0.95 | 0.23 | 0.10 | 0.19 | 0.44 |

**Additional File: Figures 1 & 2 – Predictions vs Measured concentrations using the proportional and nearest monitor approach.**

**
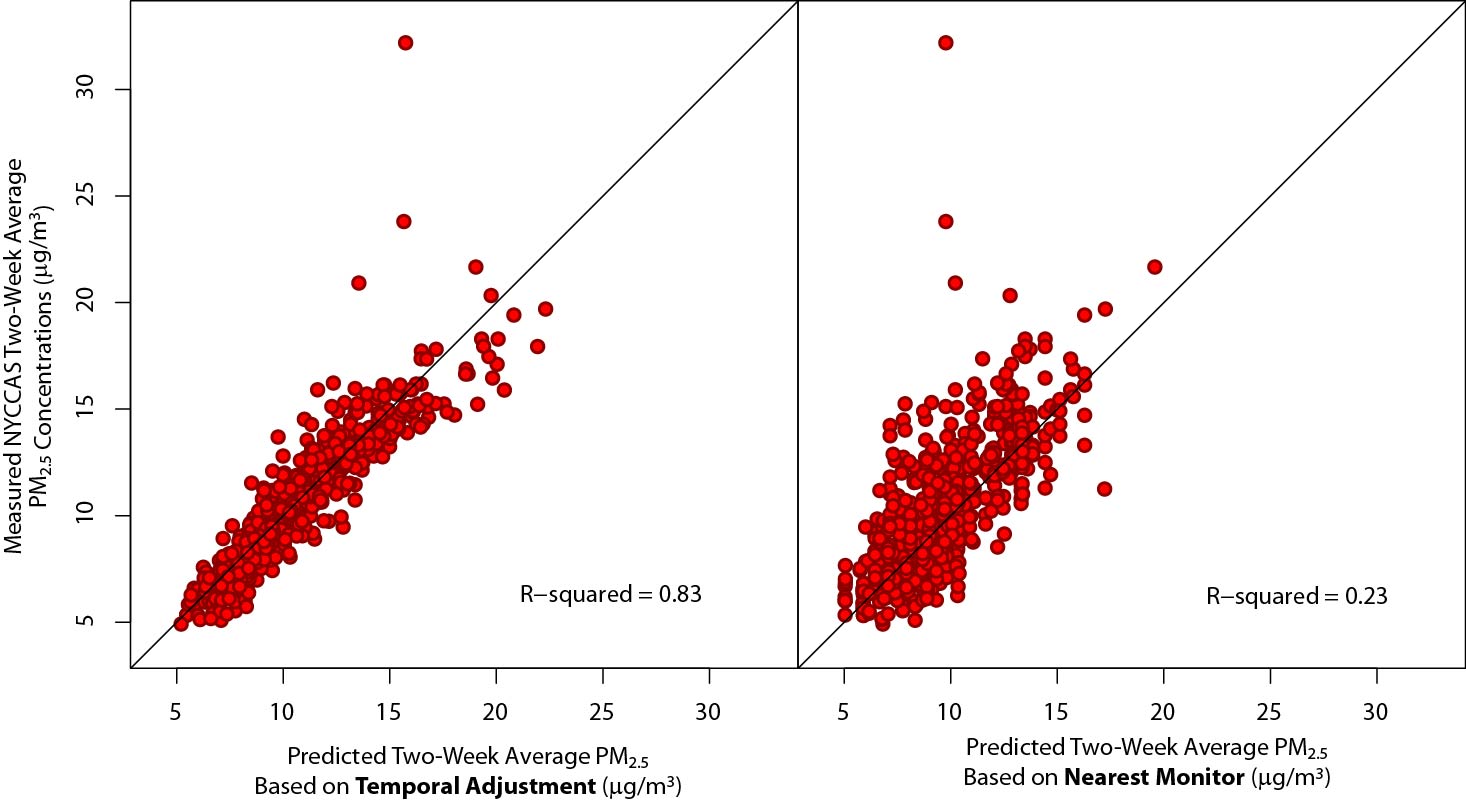
**

**
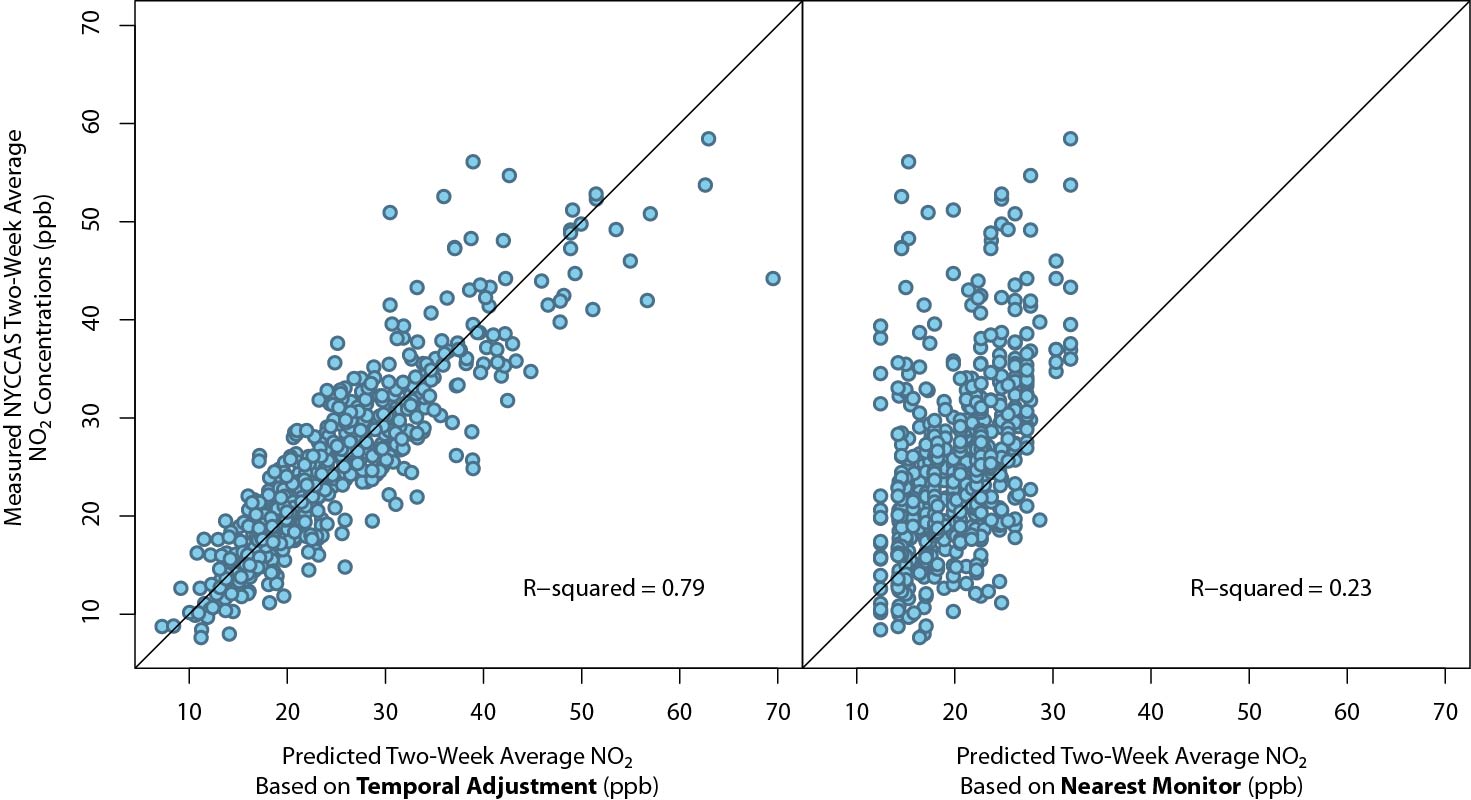
**
